# Medical Classes

**Published:** 1845-11

**Authors:** 


### MEDICAL CLASSES.

One cannot walk the streets of Philadelphia, at the present time, without being struck with the multitude of strange faces to be seen at every square and in every street. Residents, readily discover in the genteel dress and youthful appearance of the strangers that they belong to our annual class of visitors to the Medical Schools of this city. Judging from the number of students to be seen in the streets and at the Clinics, we infer that the classes of the approaching session will be larger than any that have ever assembled here before. Numerous however as they may be, they will find ample accommodations of the best character, and a hearty welcome from the respectable inhabitants with whom they may have the opportunity or occasion to become acquainted.

